# Using diphenyleneiodonium to induce a viable but non-culturable phenotype in *Mycobacterium tuberculosis* and its metabolomics analysis

**DOI:** 10.1371/journal.pone.0220628

**Published:** 2019-08-01

**Authors:** Amar Yeware, Suwarna Gample, Sonia Agrawal, Dhiman Sarkar

**Affiliations:** 1 Academy of Scientific and Innovative Research (AcSIR), Ghaziabad, India; 2 Combi Chem Bio Resource Center, Organic Chemistry Division, National Chemical Laboratory, Pune, Maharashtra, India; Infectious Disease Research Institute, UNITED STATES

## Abstract

Depletion of oxygen levels is a well-accepted model for induction of non-replicating, persistent states in mycobacteria. Increasing the stress levels in mycobacterium bacilli facilitates their entry into a non-cultivable, dormant state. In this study, it was shown that diphenyleneiodonium, an inhibitor of NADH oxidase, induced a viable, but non-culturable state in mycobacteria, having similar features to dormant bacilli, like loss of acid-fastness, upregulation of stress-regulated genes and decreased superoxide levels as compared to actively growing bacilli. Comprehensive, untargeted metabolic profiling also confirmed a decrease in biogenesis of amino acids, NAD, unsaturated fatty acids and nucleotides. Additionally, an increase in the level of lactate, fumarate, succinate and pentose phosphate pathways along with increased mycothiol and sulfate metabolites, similar to dormant bacilli, was observed in the granuloma. These non-cultivable bacilli were resuscitated by supplementation of fetal bovine serum, regaining their culturability in liquid as well as on agar medium. This study focused on the effect of diphenyleneiodonium treatment in causing mycobacteria to rapidly transition from an active state into a viable, but non-cultivable state, and comparing their characteristics with dormant phenotypes.

## 1. Introduction

*Mycobacterium tuberculosis* (Mtb) is a pathogen that develops latent phenotypes inside the host and causes tuberculosis (TB). A literature survey revealed that induction of a dormant or non-replicating persistent state in mycobacteria might be due to various kinds of stress such as oxygen limitation, nutrient starvation and multiple stresses [1–3]. The dormant form of Mtb cells can persist for many years inside the host, causing latent phase TB, before their transition into actively dividing bacilli and development of active TB symptoms, which is a major challenge for clinical treatment [4]. Two dormancy phenomena have been described in non-sporulating bacteria: the viable but non-culturable (VBNC) state, and bacterial persistence [5]. Additionally, the Cornell model, a long post-stationary phase, and potassium deficiency model have been reported in the VBNC state of Mtb [4, 6–7]. Furthermore, it has also been observed that persistent populations of Mtb in sputum can grow in liquid but not on solid culture media [8]. Persistent organisms are in a state of dormancy, defined as ‘a reversible state of low metabolic activity, in which cells can persist for extended periods without division’ [9]. In addition, treatment with antibiotics in Mtb-infected mice showed that the number of culturable cells extracted from organs had ‘zero’ colony-forming units (CFU) [10]. Therefore, the formation of a VBNC state after a few days of drug treatment has raised attention in mycobacterial pathogenesis [10]. However, understanding of persistent mycobacteria is still insufficient, mainly due to the presumably very low numbers of such organisms and the lack of clear indication of their precise localization in the living tissue of the host [11]. Thus, there is a need to understand the knowledge gap between the transition of Mtb from the dormant to the VBNC state and its subsequent resuscitation on media.

Decreased oxygen levels in dormancy have been well established with greater acceptance of *in vitro* models, as their characteristics resemble the latent bacilli present in host tissues, i.e., granulomas [12–13]. Ascorbic acid (10 mM), an antioxidant also reported to induce dormancy and VBNC phenotypes in Mtb [14–15], indicates the role of reactive oxygen species (ROS) scavenging during the development of a dormant form. Superoxide, a reactive oxygen species generated during electron transfer reactions in NADH oxidase, can be inhibited by diphenyleneiodonium (DPI) [16]. Yeware et al. (2017) have described how DPI inhibits superoxide production as well as growth of *M*. *smegmatis*; this inhibition is reversed by the addition of an external source of superoxide [16]. In contrast, nitric oxide (NO) and hydrogen peroxide have been shown to be either bacteriostatic or infective growth inhibitors at moderate concentrations [17]. These results all suggest that changes in free radical levels could be responsible for the transition of Mtb from a dormant state into a VBNC state.

Dormancy is defined by loss of acid-fastness, upregulation of stress response genes, decrease in size and developing drug resistance [1, 3, 15, 18]. The transition from an active state into a VBNC state shares similar features, along with the observation of non-cultivability in liquid or solid medium and live-dead analysis [19]. In this study, it was found that DPI, an inhibitor of NADH oxidase, decreased superoxide levels and induced a VBNC state in Mtb. DPI-treated bacilli were also found to have similar characteristics to the dormant state reported earlier [18]. Supplementation of fetal bovine serum (FBS) was found to induce resuscitation of the bacilli in liquid medium, as well as on agar medium, even after ten days of DPI treatment. The untargeted metabolic data also suggested that accumulated organic acids and NADH were diverted to the production of lactate by a fermentative mode. Therefore, this study revealed that DPI treatment caused the rapid transition of Mtb from an active state into a VBNC state, which showed similarities with dormant phenotypes.

## 2. Material and methods

### 2.1 Cultures and growth conditions

All chemicals were purchased from Sigma-Aldrich, USA and Dubos medium was purchased from DIFCO, USA. *M*. *tuberculosis* H37Ra (ATCC 25177) was obtained from the Microbial Type Culture Collection (MTCC; Chandigarh, India) and sub-cultured in Dubos broth containing 5% glycerol and 10% ADC (albumin dextrose catalase supplement). *M*. *tuberculosis* H37Ra RFP strain was generated by transforming pCHERRY3 plasmid (Addgene USA) as described previously [20]. The stock was maintained at -70°C and sub-cultured once in a liquid medium before inoculation in experimental culture medium at 37°C with shaking at 150 rpm on an orbital shaker (Thermo Electron Model No.131 481; Thermo Electron Corp., Marietta, OH).

### 2.2. Effect of DPI on growth

Various concentrations of DPI were mixed with 1.5 mL of 0.35 OD_600 nm_ cultures of H37Ra RFP bacilli in the 24-well microplate. The plates were further incubated statically at 37°C and fluorescence was measured by taking 200 μL of culture into the black well plate at 587/610 nm excitation/emission (ex/em), periodically. To determine the effect on CFU at different time points, DPI-treated cultures were 10-fold serially diluted and plated on a Middlebrook 7H11 agar plate. CFU was measured according to the standard protocol after incubation at 37°C in the CO_2_ incubator.

### 2.3. Detection of viability

A Live/dead BacLight Bacterial Viability Kit (Invitrogen) was used to determine the viability of mycobacteria according to the manufacturer’s instructions. Briefly, at different time points, 200 μL of DPI-treated (4 μg/mL) H37 Ra cultures were taken in a matrix 96-well plate and a mixture of SYTO 9 (0.91 mM) and propidium iodide (PI) (14.5 mM) were added [SYTO 9:PI = 1:16; ratio was standardized for Mtb, following the manufacturer's instructions]. After 15 min of incubation, fluorescence was measured in the dark at 470 nm excitation and 530 and 630 nm emission. The live/dead cell percentage was calculated from the standard plot of percentage live versus dead at the ratio of 530/630 nm. For imaging, a mixture of dyes were added to the treated culture and incubated for 15 min. After incubation, cells were washed with PBS and resuspended in fresh medium. The smear was prepared with 10 μL of cell suspension on a grease-free slide and fluorescence imaging was performed by EVOS microscope (Life Technology, Germany).

### 2.4. Acid-fast characterization

Approximately 20 μL of DPI-treated (4 μg/mL) Mtb H37 Ra culture at different time points was evenly spread as a thin smear on a glass slide, quickly heat-fixed by flame and cooled to room temperature before staining. Fluorescent, acid-fast staining dye Auramine-O was used in combination with neutral, lipid staining dye Nile Red (9-diethylamino-5H-benzo[a]phenoxazin-5-one) as previously described [3].

### 2.5. ROS and superoxide measurements

ROS measurement in Mtb cells was performed using fluorescent dye dichlorodihydrofluorescein diacetate (DCFH-DA), following a previously reported method [21]. Briefly, 200 μL of DPI-treated (4 μg/mL) Mtb H37 Ra culture was transferred to a 96-black well plate (matrix) to which DCFH-DA (20 μM) was added and incubated at 37°C for 30 min in the dark. Fluorescence was then measured at 485/535 nm ex/em using an M5e multimode plate reader (Molecular Devices, USA).

To determine comparative levels of superoxide production under different dormancy conditions, the dihydroethidium (DHE)-HPLC method was used [16]. Briefly, DHE (50 μM) was added to 17.5 mL of the following cultures: untreated aerobically growing culture (~0.35 OD_600 nm_), DPI treated culture (4 μg/mL, 24 h), Vitamin C treated culture (10 mM, 24 h) and Wayne hypoxic dormant culture (16 days) in 20 mm × 125 mm tubes and incubated for 90 minutes in the dark. To maintain hypoxia in the Wayne tube, DHE was added by syringe through the rubber septa. After incubation, 2 mL of culture from each set of conditions was used for the extraction and estimation of 2-hydroxyethidum (2-EOH) according to the previously reported method [16].

### 2.6. Effect on respiration

The effect on NADH dehydrogenase/ electron transport chain (ETC) complex I was investigated using the XTT (2,3-bis-(2-methoxy-4-nitro-5-sulfophenyl)-2H-tetrazolium-5-carboxanilide) reduction assay as described previously [22]. The activity of Complex IV was determined using the INT (2-(4-iodophenyl)-3-(4-nitrophenyl)-5-phenyl-2H-tetrazolium) dye reduction test as described previously [23]. Briefly, at various time points, 200 μL of DPI-treated culture was mixed with INT (400 μM) in a 96-well plate. The concentration of INT was determined by a separate dose-response curve obtained with actively growing culture as the control. Absorbance was measured at 495 nm after 4 h.

### 2.7. DNA isolation, RNA extraction, cDNA synthesis and qPCR

The DPI-treated Mtb cultures were centrifuged at 10,000 rpm for 10 min and the pellet resuspended in TE buffer (pH 7.5) containing lysozyme (0.01 mg/ml) Tris EDTA buffer (pH 7.2). The CTAB DNA extraction method was used for further DNA isolation [24]. Total RNA from DPI-treated (4 μg/mL) Mtb culture was isolated using the spheroplast-based method [25]. Briefly, a spheroplast solution consisting of 0.002% lysozyme, 0.006% D cycloserine, 1.4% glycine, 0.2% EDTA and 0.1% lithium chloride (wt/vol) in distilled water was aseptically added to the DPI-treated Mtb cultures on day 1, 5 and 9. After the spheroplast treatment, cells were harvested by centrifugation and then used for total RNA isolation by the TRIzol extraction method, as previously reported [25]. Next, 1 μg of total RNA was converted into cDNA using a first-strand cDNA synthesis kit (Sigma Aldrich) at 25°C for 10 min followed by incubation at 45°C for 50 min. The differential gene expression was analyzed by the SYBR green-based method of quantitative PCR (qPCR). In brief, a 1 μL aliquot of 1:10 diluted cDNA was mixed with 5 μL of SYBR green (Eurogenetics) and suitable primer/s (S1 Table). The volume was made up to 10 μL with water and a qPCR reaction was run for 40 cycles (PikoReal96, Thermo Scientific), according to the manufacturer’s instructions. The critical threshold values (Ct) of each test were used to analyse the differential gene expression along with *SigA* as the housekeeping gene.

### 2.8. Resuscitation

The Mtb H37Ra RFP culture at ~0.35 OD_600nm_ was treated with DPI (4 μg/mL) for 24–36 h, centrifuged at 2000 g for 10 min at 4°C and then resuspended in fresh Dubos albumin broth. The resuspended culture was further diluted to 1:10 and 1.5 mL aliquots distributed in a 24-well plate. FBS (Sigma Aldrich, USA/ PAN Biotech, UK) was then added to each test well of the 24-well plate on day 1, 5 and 10 of DPI treatment, and further statically incubated 37°C in the CO_2_ incubator. At various time points, 200 μL of culture was transferred to a black well plate and fluorescence was measured at 587/610 nm ex/em as an indication of growth. A Middlebrook 7H11-agar plate with suitable dilutions was used for CFU determination of the remaining sample.

### 2.9. Metabolite extraction and metabolomics profiling

*M*. *tuberculosis* cells were harvested, and metabolites extracted as previously described [26]. Briefly, 10 mL of culture at ~0.35 OD_600nm_ was quenched rapidly in 10 mL of 100% methanol at -20°C and centrifuged at 10000 rpm for 10 min. Cell pellets were resuspended in 2 mL of acetonitrile:methanol:water (4:4:2), transferred to 2 mL screw-cap tubes containing silica beads, and then agitated three times in a FastPrep-24 (MP Biomedicals) for 45 s at 4500 rpm, with 5 mins on ice between agitations. Samples were spun briefly, and the supernatant of the extract was filtered through a 0.22 μm syringe filter (Pall Life science Ltd) then frozen at -70°C before analysis.

Metabolomics analysis was performed using an Agilent 6540 UHD QTOF-MS with UPLC system (Agilent Technologies, Santa Clara, CA, USA). The flow rate was 0.5 mL/min with mobile phase A (100% acetonitrile) and mobile phase B (100% water). Both A and B contained 0.1% formic acid. The runtime was 30 min for the positive ion mode and 30 min for the negative ion mode. The raw data files were collected with .*mzdata*.*xml* format and further processed for analysis using the XCMS online program (https://xcmsonline.scripps.edu). During the data processing for all time points, the significance level was p < 0.05, fold change (FC) > 1.5 and intensity > 1000. Cloud plots of system biology results were extracted in an excel sheet, along with the list of predicted metabolites with their fold changes compared to untreated samples, from which the pairwise job was submitted. Duplicate samples of two independent experiments were run for each time point.

## 3. Results

### 3.1. Diphenyleneiodonium converts actively growing mycobacteria into viable but non-culturable state cells

**Fig 1:**
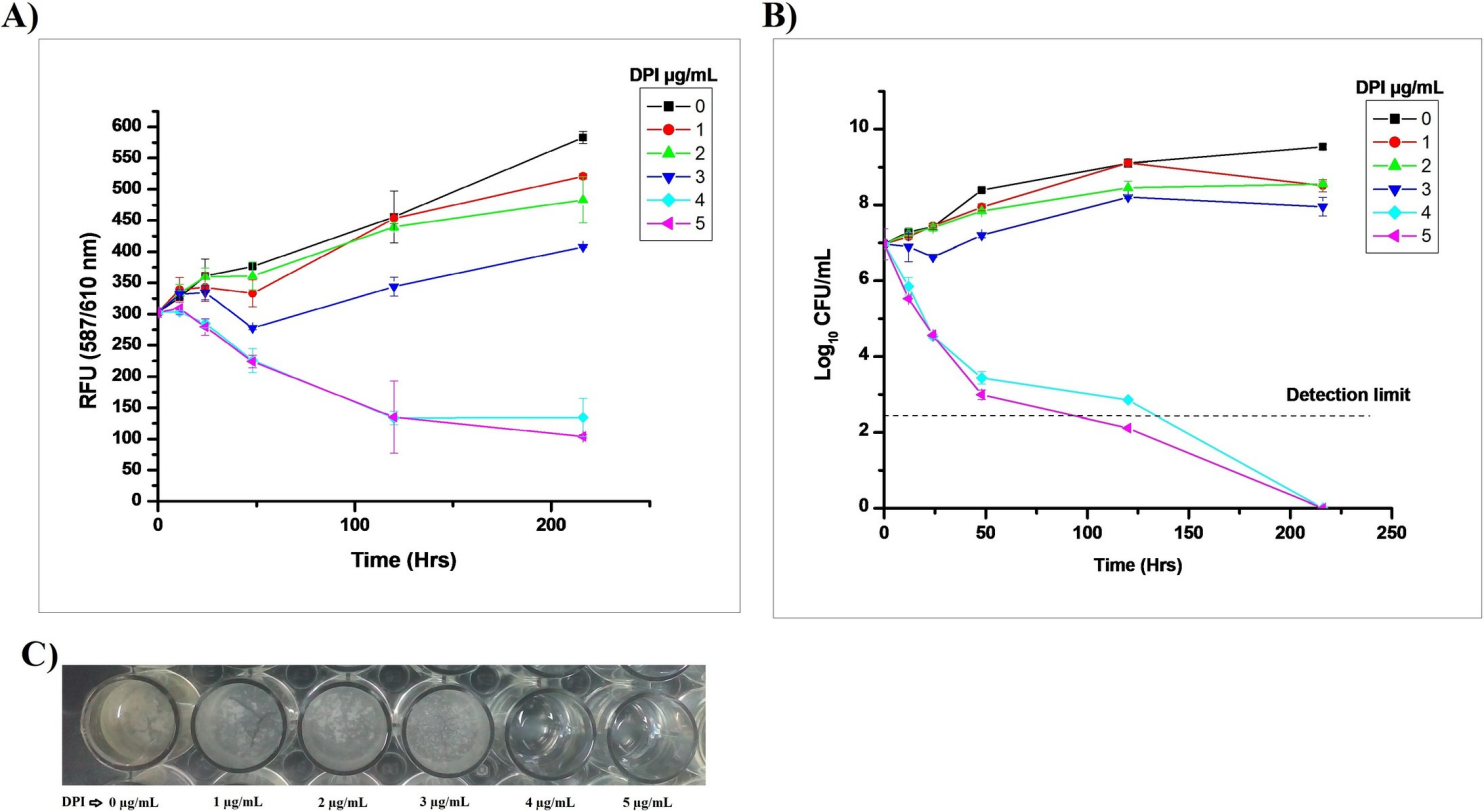
Effect of DPI on the growth of *M*. *tuberculosis*.

**Fig 2:**
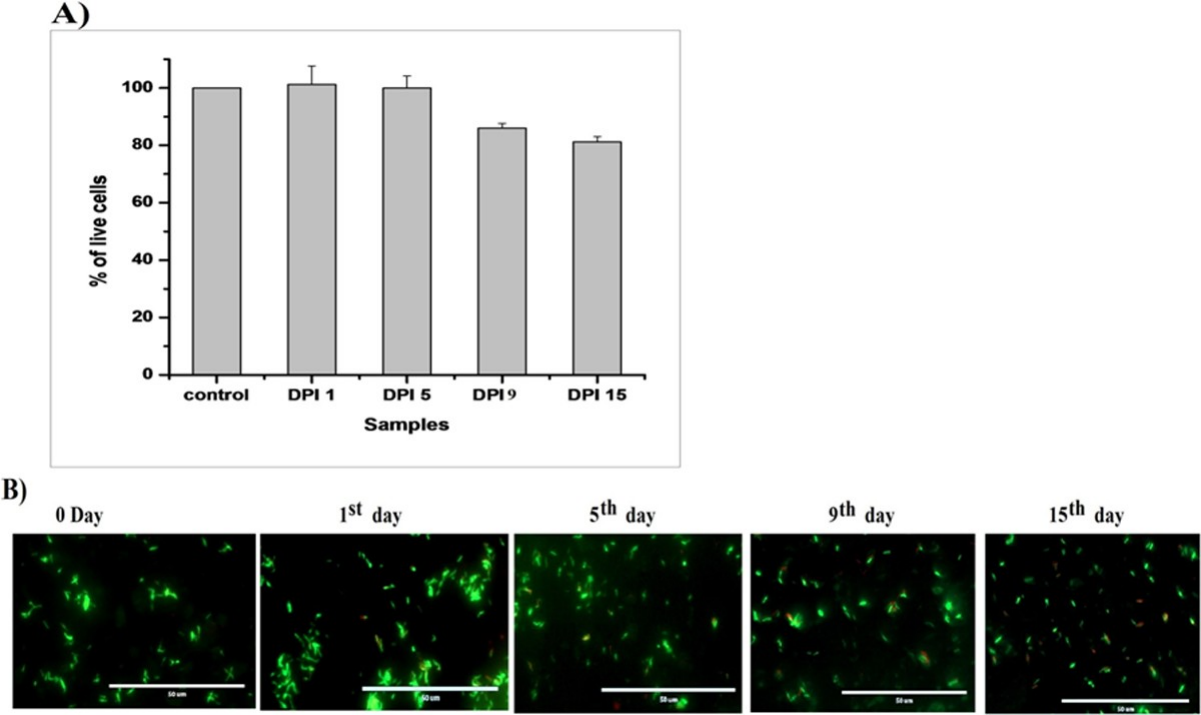
Live/dead analysis of DPI-treated *M*. *tuberculosis*. The viability of DPI-treated (4 μg/mL) Mtb cells was determined using the Live/dead BacLight Bacterial Viability Kit (Invitrogen) according to the manufacturer’s instructions. A) Percentage viability analysis carried out using a standard curve; (B) merged images of the SYTO 9 and PI staining captured by EVOS microscope as mentioned in the “Materials and Methods section.” The data shown is representative of three independent experiments (scale bar 50 μm).

During the dose-response Mtb study, > 4 μg/mL DPI concentration showed a decrease in fluorescence with time (Fig 1A). CFUs on the agar medium were also found to decrease by 3.53 ± 0.16 log_10_ CFU/mL within 48 h of treatment by >4 μg/mL DPI (Fig 1B and 1C). When the DPI-treated culture was washed twice after 24 h, diluted 1:10 in fresh Dubos medium with 10% OADC supplement, and incubated with continuous shaking at 37°C, no growth was evident compared to untreated bacilli (S1 Fig). DPI-treated bacilli were subjected to SYTO 9 and propidium iodide (PI) staining, which showed 100% viability up to 5 days then slightly decreasing by ~15% within 9–15 days of treatment (Figs 2 and S2). The stability of DPI was also estimated by HPLC, revealing that ~1.5 μg/mL of DPI was depleted by Mtb after 3 h of treatment, whereas > 3.5 μg/mL of DPI was always present in the supernatant for up to 9 days when using 5 μg/mL of DPI for treatment (S3 Fig).

Various concentrations of DPI (1–5 μg/mL) were added to ~0.35 OD_600 nm_ of growing Mtb H37 Ra RFP strain. A 200 μL aliquot of treated culture was transferred to a black well plate and fluorescence was measured periodically at 587/610 nm ex/em. The CFUs were determined from the remaining sample with suitable dilutions on a Middlebrook agar 7H11 plate and incubated at 37°C for one month. The data is shown as the mean of triplicate results with ±SD and reproducibility determined at least two times. A) Fluorescence reading at ex/em 587/610 nm; B) CFU count; and C) Mtb growth monitored at different concentrations of DPI.

### 3.2. DPI treatment influences dormant continuum in *M*. *tuberculosis*

**Fig 3:**
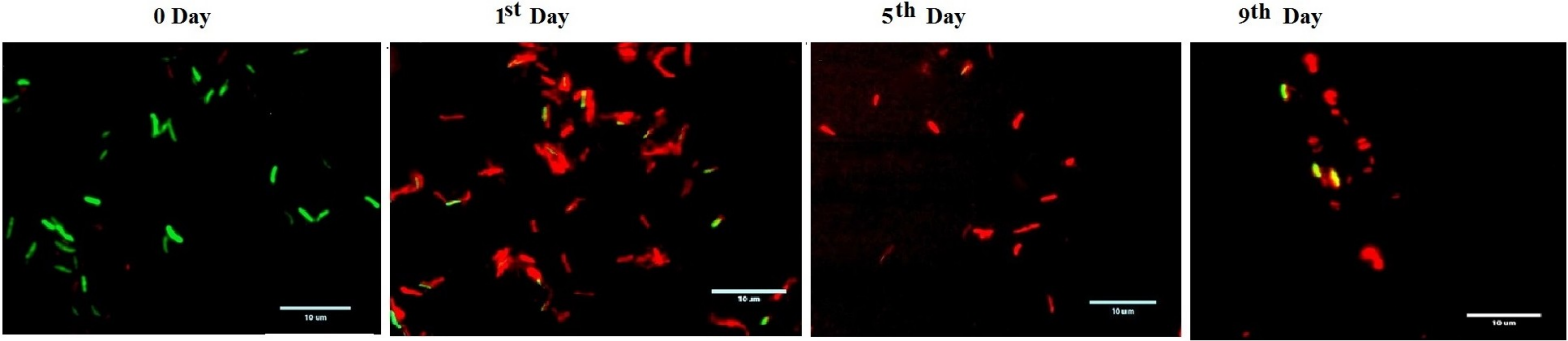
Acid-fast staining of DPI-treated *M*. *tuberculosis*. Acid-fast staining of DPI-treated (4 μg/mL) culture using Auramine O-Nile Red at respective time points by preparing a smear on a glass slide. The images were captured by EVOS microscope with an oil immersion (100X) objective lens and processed further using Microsoft Paint software (scale bar 10 μm).

**Fig 4:**
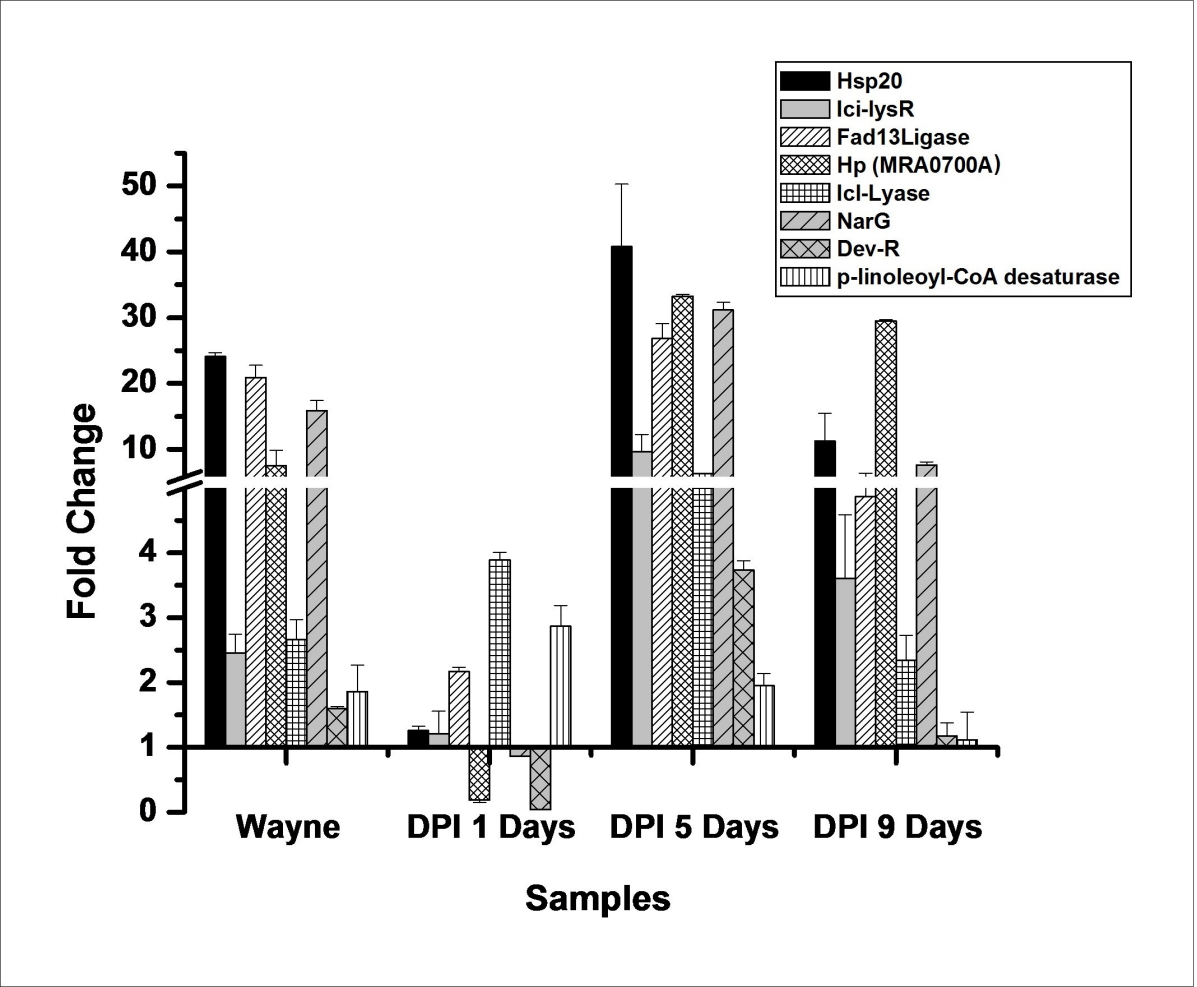
Differential gene expression analysis of DPI-treated *M*. *tuberculosis*. The total RNA was extracted and converted into cDNA from the DPI-treated Mtb cultures from 1 day- (D1), 5 day- (D5), 9 day- (D9) and 16 day-old Wayne hypoxia models (W). Quantitative estimation was performed using the SYBR green-based qPCR kit and fold change was calculated for each gene by normalizing with *SigA* as a reference. The data is represented as the mean of three identical experiments ±SD (p < 0.05).

Dormancy is well characterized, with loss of acid-fastness and upregulation of stress-regulated genes [3, 18, 27]. A similar phenotype was observed for Mtb bacilli, with loss of acid-fastness after 24 h of DPI treatment upon staining with Auramine O and Nile Red (Fig 3). Additionally, upregulation of the genes *Hsp20*, *Ici-lysR*, *Fad13Ligase*, *Hypothetical protein (MRA0700A)*, *Icl-Lyase*, *NarG* and *Dev-R* at 40.78, 9.65, 26.86, 33.26, 6.32, 31.13 and 3.73-fold, respectively, was observed on day 5 of DPI treatment compared to untreated bacilli (Fig 4) [2–3, 28], and *Dev-R*, *Icl-Lyase* and putative linoleoyl-CoA desaturase (*MRA3270)*, related to fatty acid metabolism, were found to be significantly upregulated (> 2 fold) after 24 h of DPI treatment. Interestingly, although down regulation of *MRA3270* was shown in the reported dormancy model [2], this was not observed in the Wayne model or in DPI-treated bacilli (Fig 4).

**Fig 5:**
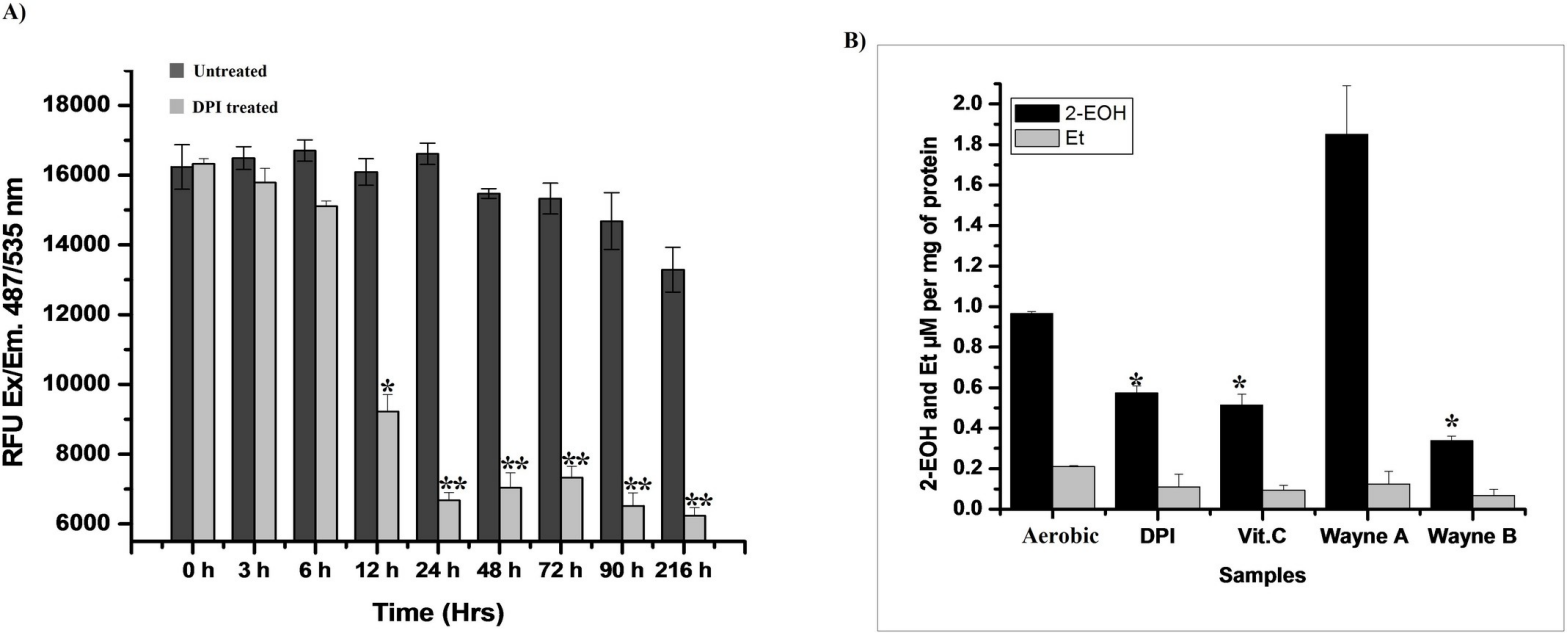
ROS and superoxide decrease in the dormant form of mycobacteria. **A) ROS estimation:** A 200 μL aliquot of DPI-treated (4 μg/mL) culture was periodically transferred to a 96-black well plate (matrix), to which 20 μM of DCFH-DA was added and incubated at 37 ^o^C for 30 min in the dark. Fluorescence was measured at 485/535 nm ex/em. **B) Superoxide estimation:** 50 μM of DHE was added to 17.5 mL of DPI-treated, 10 mM of Vit.C-treated (24 h), and 16 day-old Wayne hypoxia culture (Wayne A: exposed to air; Wayne B: hypoxic conditions) and incubated for 90 min. Levels of 2-EOH and ethidium (Et) were determined. The data is shown as the mean of the three identical results ±SD (*p < 0.05, **p < 0.005).

Fluorescent dye DCFH-DA was used to determine the total ROS level. A 56% decrease in the relative fluorescence unit (RFU) compared with untreated Mtb bacilli were observed within 24 h of DPI (4 μg/mL DPI) treatment (Fig 5A). Furthermore, 2-hydroxyethidum (2-EOH), a specific product formed during the reaction of superoxide with DHE, was found with values of 0.924 ± 0.16, 0.479 ± 0.06, 0.391 ± 0.09 and 0.49 ± 0.07 μM per mg of protein in actively growing, DPI-treated (24 h), 10 mM Vitamin C-treated (24 h) and 16 day-old Wayne hypoxic cultures, respectively (Fig 5B). Interestingly, the level of 2-EOH was found to be 1.23 ± 0.14 μM per mg of protein when DHE was incubated under aerated conditions in 16 day-old Wayne hypoxic Mtb cultures. This indicated that a rapid burst of superoxide occurs in hypoxic Mtb when it is oxygenated (Fig 5B).

### 3.3. Effect on respiration and DNA fragmentation

**Fig 6:**
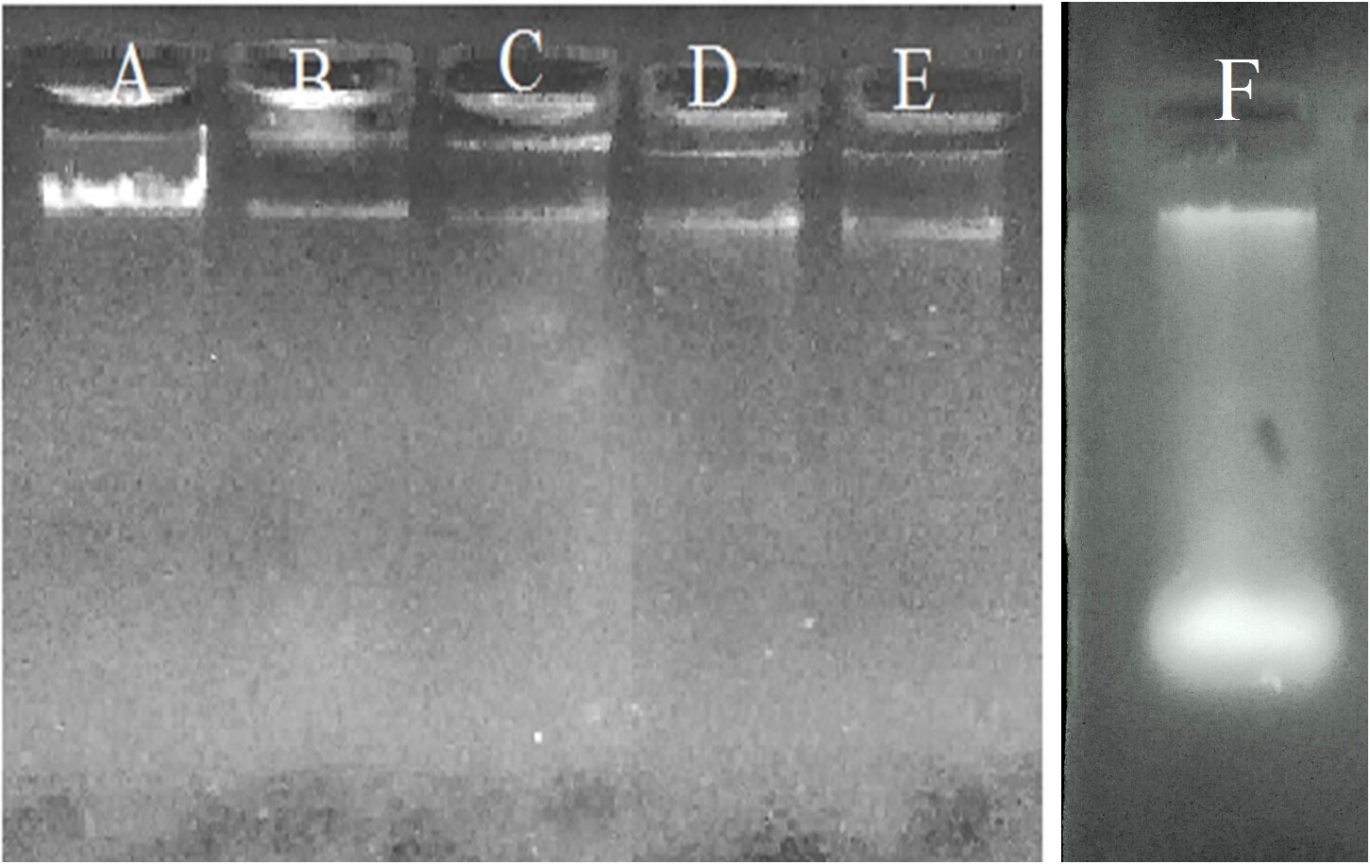
DNA extraction from the DPI-treated *M*. *tuberculosis*. DNA was extracted from actively growing and DPI-treated (4 μg/mL) Mtb samples and run on 1% agarose gel. A) Actively growing culture; B-E: 1^st^, 5^th^, 9^th^ and 15^th^ day DPI-treated cultures, respectively; F: UV-treated Mtb culture).

The ETC is the major process associated with respiration of any live cell. Cellular activity was determined using tetrazolium dyes in culture suspension. The absorbance of XTT {2,3-Bis-(2-Methoxy-4-Nitro-5-Sulfophenyl)-2H-Tetrazolium-5-Carboxanilide)} or INT {2-(4-iodophenyl)-3-(4-nitrophenyl)-5-phenyl-2H-tetrazolium} dye’s reduction was found to be decreased (< 0.2 OD) when they were incubated with DPI-treated bacilli compared to untreated Mtb bacilli (0.81 ± 0.02 and 0.52 ± 0.01 at 470 nm and 490 nm, respectively) (S4A and S4B Fig). Intact DNA was observed in each lane of the agarose gel electrophoresis, representing samples isolated from the 1^st^, 5^th^, 10^th,^ and 15^th^ day of DPI-treated Mtb bacilli (Fig 6), which indicated that DPI treatment did not cause DNA fragmentation; it retained its stability.

### 3.4. Resuscitation of non-cultivable *Mycobacterium tuberculosis* using fetal bovine serum

**Fig 7:**
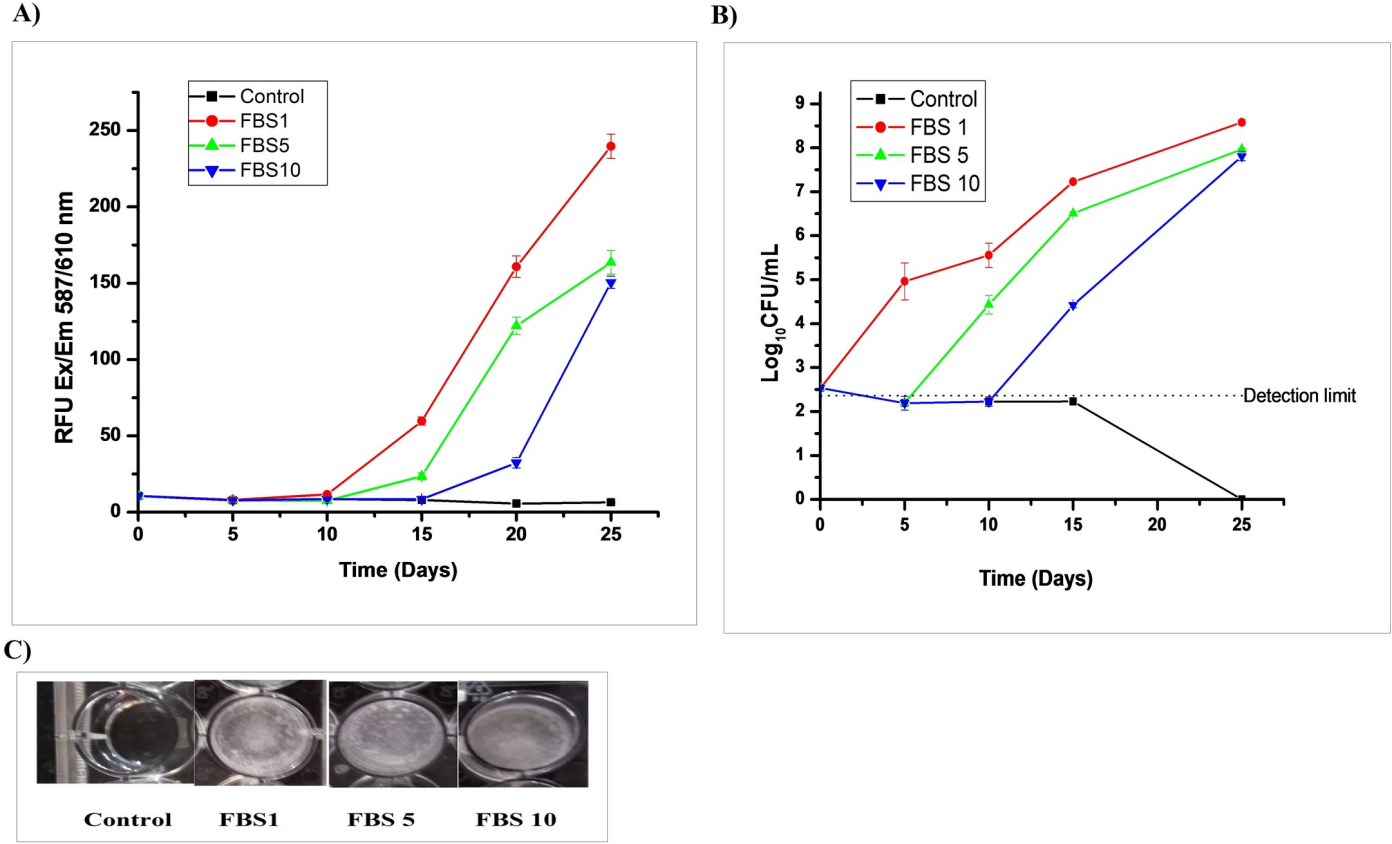
Resuscitation of DPI-treated bacilli by fetal bovine serum. The mycobacterial culture was treated with 4 μg/mL of DPI (OD_600 nm_ = 0.35) for 24–36 h, washed and resuspended in fresh Dubos albumin broth. The culture was further diluted 1:10 in the Dubos medium and distributed in 24-well plates as 1.5 mL aliquots. FBS (20–30 μL) was added to the 24-well plates on days 1, 5 and 10 of incubation of washed DPI treated bacilli. A) The fluorescence, and B) number of CFUs were determined after every 5^th^ day of FBS supplementation. C) Images of Mtb growth in wells on days 1, 5 and 10 of FBS supplementation to washed DPI treated bacilli were captured at the endpoint incubation (control: without FBS). The results are shown as the mean of triplicate results ±SD. The reproducibility of the experiment was determined two times.

VBNC cells may be resuscitated back to culturable cells under suitable stimuli [19]. However, as DPI treatment decreased the superoxide levels in Mtb, no reverse effect was seen after the addition of an external source of superoxide, i.e., pyrogallol and menadione (S5 Fig). Despite this result, supplementation of the DPI-treated Mtb with FBS resulted in significantly increased growth (> 5 log_10_ CFU/mL) in the VBNC state culture within 10 days (Fig 7).

### 3.5. Metabolic profile of DPI-treated *M*. *tuberculosis*

Untargeted metabolite analysis was done on DPI-treated (4 μg/mL) mycobacteria at various time points using XCMS online software suite including the pairwise, meta and multi-group data analysis functions [29–31]. Untreated culture from the same batch was used as the control.

**Fig 8:**
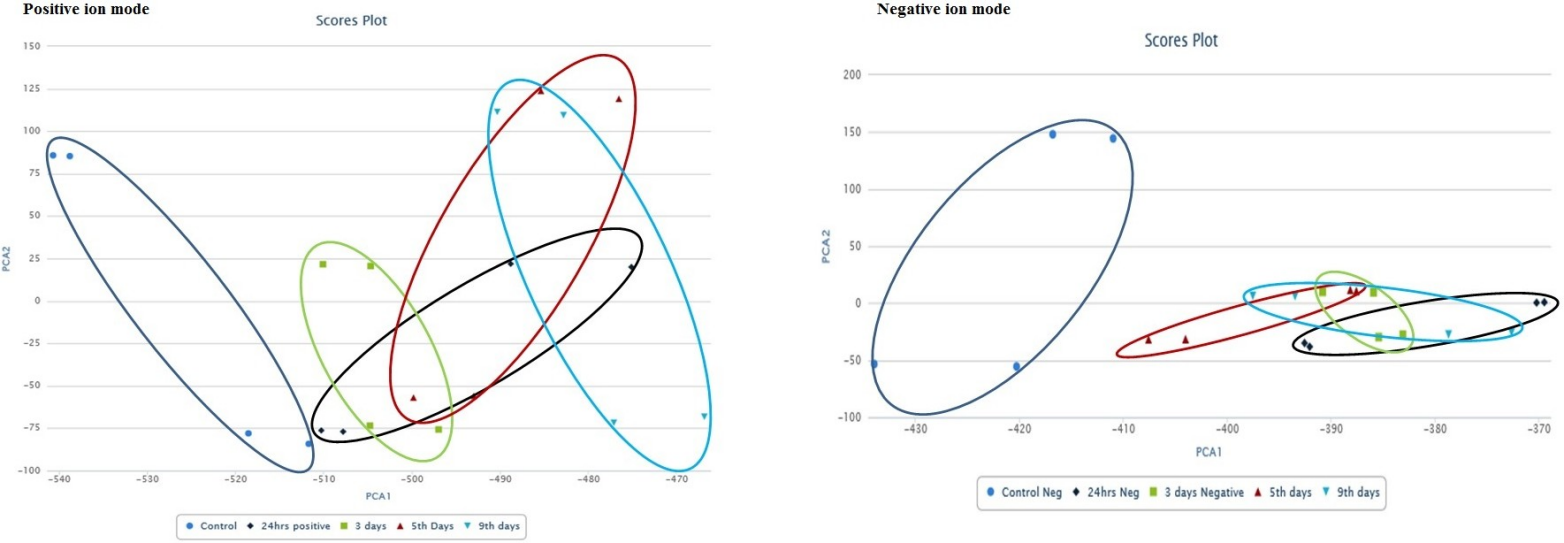
Interactive principal component analysis score plot. A) Positive ion mode; B) negative ion mode. Multi-group mode was selected to obtain the PCA for all samples in XCMS online suits (https://xcmsonline.scripps.edu). Data is representative of duplicate samples of two biologically-independent experiments.

The unit variance between the control and treated group was determined by providing all the data points with intensity to multi-group inputs, and score principal component analysis (PCA) plots were generated (Fig 8). Fig 8A represents the ~1000 feature coverage for control versus a treated group of positive ion modes where 0.927 and 0.932 unit variance (R^2^ value) was observed for PC 1 and PC 2, respectively. Multi-group XCMS analysis of negative ion mode metabolite PC 1 and PC 2 unit variance was observed to be 0.917 and 0.934, respectively (Fig 8B). Therefore, the PCA scores revealed that metabolomes of the DPI-treated and untreated samples differed from each other at all time points (Fig 8).

Additionally, changes in levels of commonly dysregulated metabolites were determined using Meta-XCMS [30]. 108 and 159 common features were observed in positive and negative ion modes, respectively, and at all time points had p < 0.05, FC >1.5 and intensity >1000 (S6 Fig).

**Fig 9:**
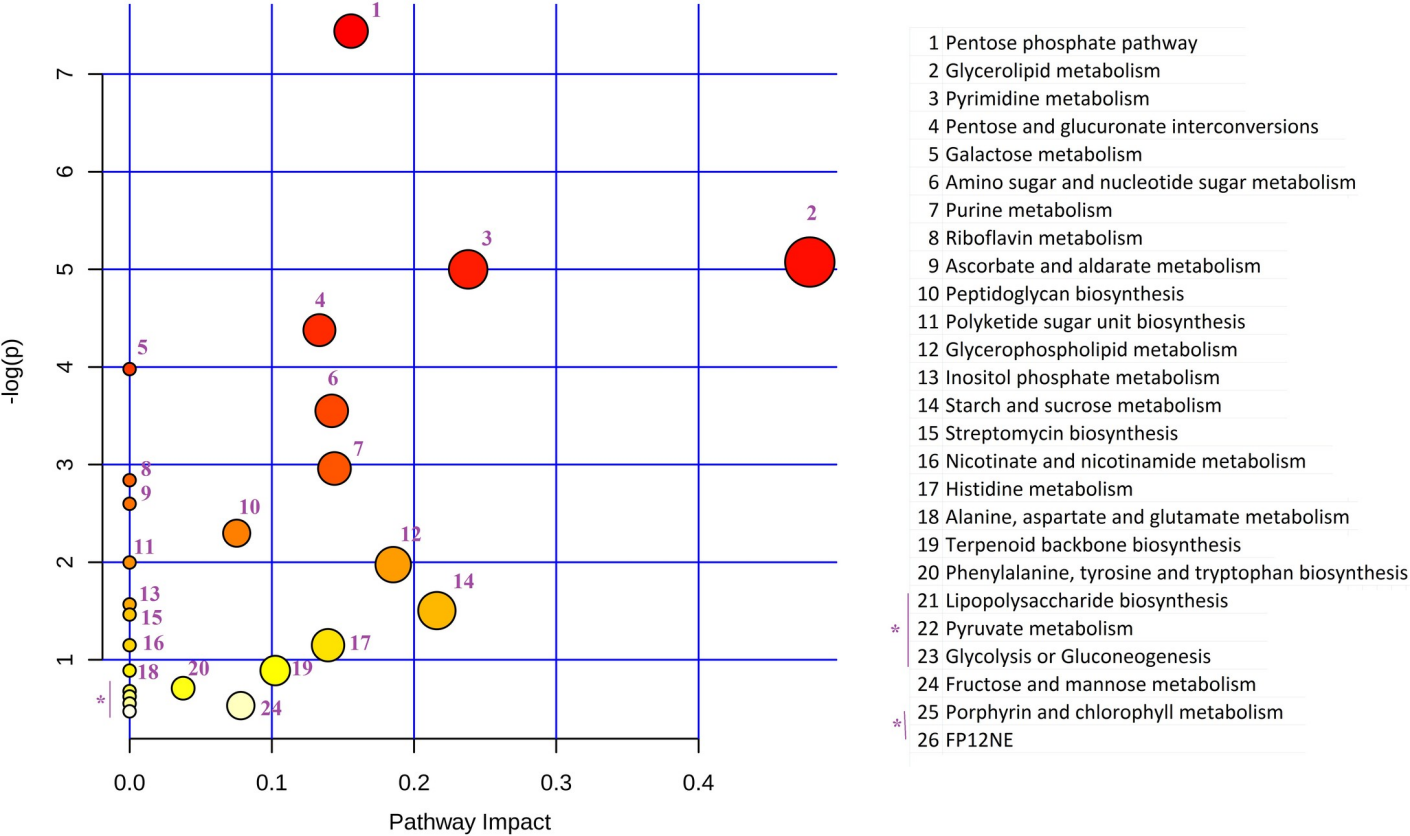
Altered pathway impact analysis. Metabolites predicted by the XCMS pairwise job for each time point were compiled in a single file, and common metabolites were sorted and processed using MetaboAnalyst for pathway impact analysis using the prokaryotic system. (http://www.metaboanalyst.ca/faces/upload/PathUploadView.xhtml). Color variation from yellow to red indicates the increased levels of significance of metabolites in the data, and size of bubbles indicates their impact.

To investigate which metabolites were most responsible for the observed differences between control (untreated) and treated groups, pairwise comparison of control samples and treated samples for each time point was performed using the XCMS online tool [29]. The altered metabolite levels were obtained in the form of a cloud plot for both positive and negative ion modes with p < 0.05 and FC > 1.5 (S7A and S7B Fig). Furthermore, system biology results were used to prepare a predicted metabolite heat map, and pathway impact analysis was performed using MetaboAnalyst software (Fig 9). Overall, ~103 dysregulated compounds were predicted during the transition at all four time points of DPI treatment (S8 Fig).

Metabolites involved in nucleotide metabolism like adenine, dTMP and CMP were decreased > 2 fold during treatment, while pentose sugars D-ribose-1-phosphate, D-ribulose 5-phosphate and D-xylulose 5-phosphate were observed to increase during treatment along with the key metabolites of purine metabolism, hypoxanthine and RNA component UTP (S8A Fig). Of the organic and other amino acid metabolites, L-argininosuccinate, 2-aminoprop-2-enoate (L-alanine), 2-iminobutanoate and (2Z)-2-aminobut-2-enoate significantly decreased (FC > 2) at all time points, while fumarate and succinate accumulated on day 9 of DPI treatment, showing strong disruption of the alanine, aspartate, glutamate and tricarboxylic acid cycle (TCA) pathways [32] (Fig 9). Furthermore, components of the TCA cycle like citrate and isocitrate (*cis*-vaccenate) were decreased along with malate, indicating the continued utilization of these products via glyoxylate shunt along with further accumulation of succinate or acetolactate/lactate via pyruvate metabolism (S8B and S8C Fig).

On day 3 of DPI treatment, it was observed that meso-diaminopimelate and L,L-diaminopimelate were significantly increased. This indicated increasing rigidity of the cell wall during treatment, as reported for dormancy phenotypes [19]. However, UDP-N-acetyl-α-D-glucosamine and UDP-α-D-glucose/galactose were found to decrease during treatment, which could be due to building blocks not being synthesized during maintenance of a non-culturable state. Additionally, as described above regarding nucleotide metabolism, pentose sugars D-ribose-1-phosphate, D-ribulose 5-phosphate, D-xylulose 5-phosphate and D-sedoheptulose 7-phosphate increased on day 1 of treatment, highlighting the importance of pentose phosphate pathway (PPP) activation after treatment [33] (S8D Fig). Palmitoleate and L-1-glycero-3-phosphocholine, which are lipid precursors, were found to decrease at three time points during DPI treatment (S8E Fig). Furthermore, sulfur-containing metabolites, reported to influence bacterial pathogenesis [34], were also altered during DPI stress. Mycothiol (MSH), the primary thiol-containing small molecule of mycobacteria, increased on day 1 of treatment and decreased on day 3 (S8E Fig), and sulfate, myo-inositol and glutathioselenol increased at the early time points. Conversely, mycothoine, dethiobiotin and L-cystathionine were observed to decrease at more than two time points after DPI treatment. Nicotinate and NAD decreased on day 1 of treatment, indicating NADH oxidase inhibition by DPI (S8G Fig).

In summary, the metabolite profiles subjected to the pathway impact analysis showed that alanine, aspartate and glutamate metabolic pathways were highly altered and ultimately affected nitrogen metabolism under DPI-treated conditions (Fig 9). On the other hand, in energy-related pathways (i.e., TCA and pentose phosphate), metabolites showed increased levels or accumulation during DPI treatment. Furthermore, several macromolecular building blocks (e.g., amino acids, fatty acids, purine, etc.) and cytoskeletal molecules like peptidoglycan and lipids were found to be altered during DPI treatment (Fig 9).

## 4. Discussion

The influence of oxygen on the growth of *Mycobacterium* spp. has been detailed in the literature [1, 14]. Earlier studies have shown that the level of superoxide production is proportional to the availability of oxygen in the environment [28, 35]. Superoxide has reported a contradictory effect on Mtb, ranging from lethal effects to a role in cell signaling or growth [36, 16]. DPI has been shown to inhibit superoxide production and bacterial growth, which was reversed by the addition of an external source of superoxide in *M*. *smegmatis* [16].

In this study, it was observed that DPI also inhibited the growth of Mtb and caused it to rapidly enter into a VBNC state (Fig 1). Our primary observation showed a gradual decrease in red fluorescence (Fig 1A), this could be due to either a reduced rate of protein synthesis upon DPI treatment or a transition effect to dormant bacilli. Additionally, CFU count was found to be beyond the detection limit within 48 h of treatment but viability was retained (> 83%) until day 9 of DPI treatment (Fig 2B). A small number of CFU counts were found at initial time points (i.e. 24 h and 48 h) in the DPI-treated cultures, where these subpopulations were unable to grow in Dubos broth (Figs 1B and S1). The subpopulation observed on the agar plate could have been at an intermediate stage and entered in to a VBNC state in the liquid microenvironment. Other possible reasons could be the different composition, texture, and higher oxygen level of the solid media compared to the liquid media. The slight decrease in viability may have been due to altered cell membrane permeability (bearing dormancy characteristics), decreased cell size, the effect on the ratio of STYO 9: PI, or simply that some bacilli could have died during the treatment.

Mycobacterial cells are well known for their ability to assume a VBNC state under *in vivo* or *in vitro* conditions and are mostly characterized by dormant features [4]. In the study, it was found that, after DPI treatment, Mtb transitioned to a VBNC state through dormancy (Figs 3 and 4). The Auramine O staining changed after 24 h of DPI treatment, and upregulation of the *FAD13* ligase enzyme was observed in 24 h DPI-treated cDNA samples (Fig 4). Upregulation of *HspX*, *DosR* and *Icl* genes has also been reported in various dormancy models [3, 27], which confirms the transition of these bacilli to a dormant-like state; however, *DosR* was not observed to be significantly upregulated in this study. This finding indicates the likelihood that bacilli transition occurs in normoxia (oxygenated) conditions, as reported in the Vitamin C dormancy model [14].

The capacity for superoxide production in either form of dormant bacilli was also found to be decreased compared to the actively growing bacilli (Fig 5). This observation supported the proposal that superoxide levels are probably responsible for the development of dormancy features in Mtb. XTT and INT were used to determine the functional activity of the ETC. Interestingly, the rate of reduction of these dyes was also found to be decreased in DPI-treated Mtb, indicating the shutdown of ETC or ATP generation energetics under such conditions (S4 Fig). Furthermore, DNA and RNA extraction of DPI-treated cells was found to remain intact even after day 15 of DPI treatment, indicating the viability of these cells (Fig 6). In contrast to *M*. *smegmatis* cells, the addition of menadione or pyrogallol failed to resuscitate this DPI-treated Mtb culture [16], probably due to inherent differences between these two species. Regardless, this DPI-treated culture was successfully resuscitated by supplementation of FBS in the culture (Fig 7). Further studies are being carried out to identify which active ingredient(s) present in FBS are responsible for resuscitation of a VBNC state in Mtb.

In metabolomics analysis, metabolites are considered the products of gene expression and functional regulation at the protein level [37] and can be used to give mechanistic insight into the metabolic shifts that occur during the transition of mycobacteria from an active state to a VBNC state. Lactate (R, S, and S-acetolactate) is an end product of anaerobic glycolysis [38]. In this study, increased lactate levels were observed in the VBNC state of mycobacteria and was earlier found to be induced by Mtb in lung granuloma [38] (S8C Fig). However, it is also expected that accumulation of lactate could cause cytosolic NADH to build up as a result of the inhibition of glycolysis at the level of glyceraldehyde-3-P-dehydrogenase [38], and NADH oxidase inhibition by DPI [16]. Additionally, the observed increased levels of succinate could be a sign of the diversion of isocitrate towards succinate via glyoxylate shunt as earlier reported in hypoxia-adapted Mtb [39]. Fumarate levels were also found to increase in DPI-treated Mtb, resembling the activation of reverse TCA cycles upon oxygen limitation, and maintaining standard cellular energetics in the dormant state of mycobacteria via fumarate reductase [40].

The increase in pentose sugars like D-ribose-1-phosphate and xylulose-5-phosphate indicated the shutdown of the nucleotide synthetic pathway (S8A and S8D Fig). The component D-sedoheptulose 7-phosphate, also part of the pentose phosphate pathway (HMP shunt), increased during DPI treatment, which indicated the diversion of the glycolytic pathway for adaptation of the VBNC state. The increase in meso-diaminopimelate could be due to the formation of more cross-linkages in peptidoglycan to enhance the rigidity of the cell wall during the VBNC state [19]. Homocysteine (2-iminobutanoate), a key intermediate in the synthesis of metabolites such as methionine, cysteine, SAM, SAH and adenosine [41], was found to decrease during the VBNC state. Furthermore, components of amino acid synthesis, including alanine (2-aminoprop-2-enoate) and intermediates L-argininosuccinate and L-glutamate were decreased, resulting in changes to their activities in the metabolic pathway (Fig 9). (S)-4-Hydroxy-2-oxohexanoate and (S)-2-aceto-2-hydroxybutanoate, components of valine, leucine and isoleucine biosynthesis, were also decreased in dormant bacilli. Lipid components such as L-1-glycero-3-phosphocholine, phosphocholine and palmitoleate were also decreased, affecting the synthesis of unsaturated fatty acids. Furthermore, increased levels of sulfate, MSH, and myo-inositol were found in the initial days of DPI treatment; these are supposed to protect against oxidative stress [42]. However, the decreased concentration of NAD and nicotinate because of DPI inhibition was inconsistent with the NADH/NAD ratio found in dormant states reported earlier [43]. Overall, metabolic profiling showed decreased biogenesis of amino acids, purine and pyrimidine nucleotides, and unsaturated fatty acids due to weaker metabolism during DPI treatment and mycobacterial transition into a VBNC state.

In summary, DPI treatment resulted in decreased CFU counts, red fluorescence, loss of acid-fastness and upregulation of dormancy-related genes. Decreased levels of superoxide production were also found, similar to that observed in hypoxia and Vitamin C dormancy models. However, DPI-treated bacilli retained intact DNA, RNA and > 83% live bacilli, even after 10–15 days of treatment, confirming their viability. Metabolic profiling analysis clearly indicated that the prevalence of lactate, fumarate, succinate, MSH, myo-inositol and pentose sugars increased, while biogenesis of amino acids, nucleotides and unsaturated fatty acids decreased, again in line with the dormancy features observed within granuloma during Mtb infection of human lungs [32,38]. Therefore, this report suggests that DPI induce rapid dormancy followed by a VBNC state in Mtb cells. Thus, a DPI-induced, non-culturable state could be used as a “VBNC model” for mycobacteria.

## Supporting information

S1 Fig **Growth of DPI treated culture in Dubos broth:** The continuously growing mycobacterial culture of an OD600 ~0.35 treated with the 4 μg/mL of DPI for 24–36 hrs. Treated and untreated culture washed twice, resuspended as 1:10 dilution in the fresh Dubos albumin broth supplement and incubated for at 37 oC with stirring. After the 9–10 days of incubation image were captured and processed using Microsoft paint software.
(JPG)

S2 Fig **Standardization of Live/dead assay** A) Standard plot of % live cell (percentage of live and dead cell obtained by Isopropanol treatment) versus 630/530nm ratio. B) Fluorescence reading of with treated and untreated culture at Ex. 470 and Em. 500-700nm. Assay standardization was done according to manufacture instruction (Invitrogen, Life technology).
(JPG)

S3 Fig DPI estimation by HPLC from the of DPI treated (5 μg/mL) Mtb culture. (JPG)

S4 Fig Effect on respiration of DPI treated *M*. *tuberculosis*. An aliquot of 200 μL of DPI treated bacilli periodically transferred to 96 well plates and incubated with XTT (A) and INT (B) tetrazolium salt. After 20 min incubation with XTT, 60 μM of menadione were added and plate further incubated for 40 mins. The absorbance was measured at 470 nm for XTT reduction assay or 495 nm for the INT reduction assay. Data shown as the mean of three identical results with ±SD.
(JPG)

S5 Fig Effect of the pyrogallol and menadione of DPI treated culture. The mycobacterial culture was treated with 4 μg/mL of DPI (OD 600nm = 0.35) for 24–36 h, washed and resuspended in fresh Dubos albumin broth. The various concentration of the Pyrogallol or Menadione were added and incubated for at 37 oC. At various time point growth was measured in the form of fluorescence a Ex/Em. 587/610 nm. Data shown as the mean of triplicate results with ±SD.
(JPG)

S6 Fig Venn diagram for common feature analysis in A) Positive and B) Negative ion mode analysis.
(TIF)

S7 Fig Cloud plots of altered metabolites for each time points by A) Positive and B) Negative ion mode.
(TIF)

S8 Fig Heat map of altered metabolite of DPI treated mycobacteria at 1^st^, 3^rd^, 5^th^ and 9^th^ days of treatment. (PDF)

S1 Table Primers used in this study. (DOC)
