# Supplementary figures and images for: Using diphenyleneiodonium to induce a viable but non-culturable phenotype in Mycobacterium tuberculosis and its metabolomics analysis
Source: PLoS One. 2019 Aug 1;14(8):e0220628. doi: 10.1371/journal.pone.0220628 (PMC6675104; doi:10.1371/journal.pone.0220628)

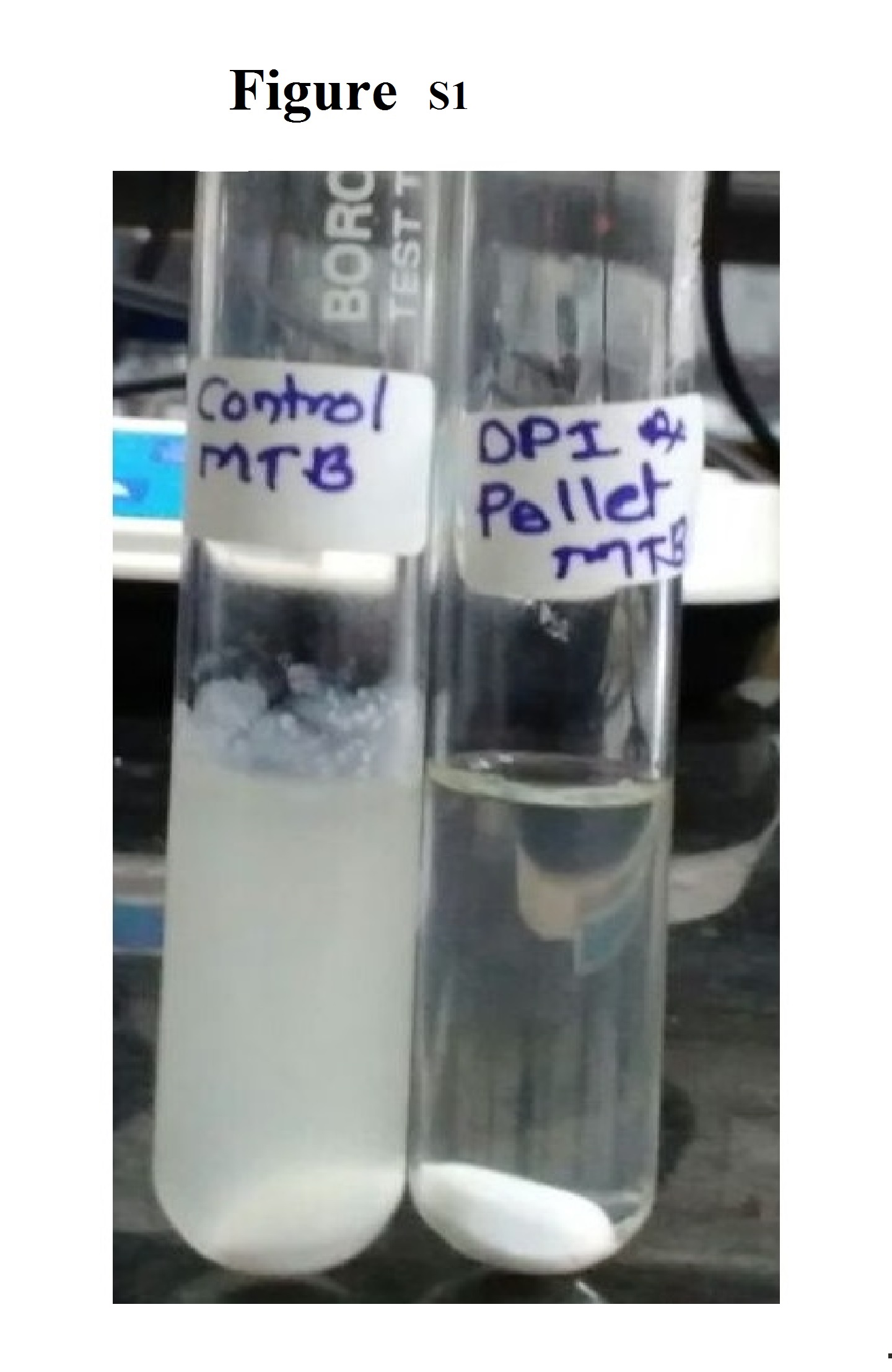

Supplement: S1 Fig — Growth of DPI treated culture in Dubos broth: The continuously growing mycobacterial culture of an OD600 ~0.35 treated with the 4 μg/mL of DPI for 24–36 hrs. Treated and untreated culture washed twice, resuspended as 1:10 dilution in the fresh Dubos albumin broth supplement and incubated for at 37 oC with stirring. After the 9–10 days of incubation image were captured and processed using Microsoft paint software. (JPG) [file pone.0220628.s001.jpg]

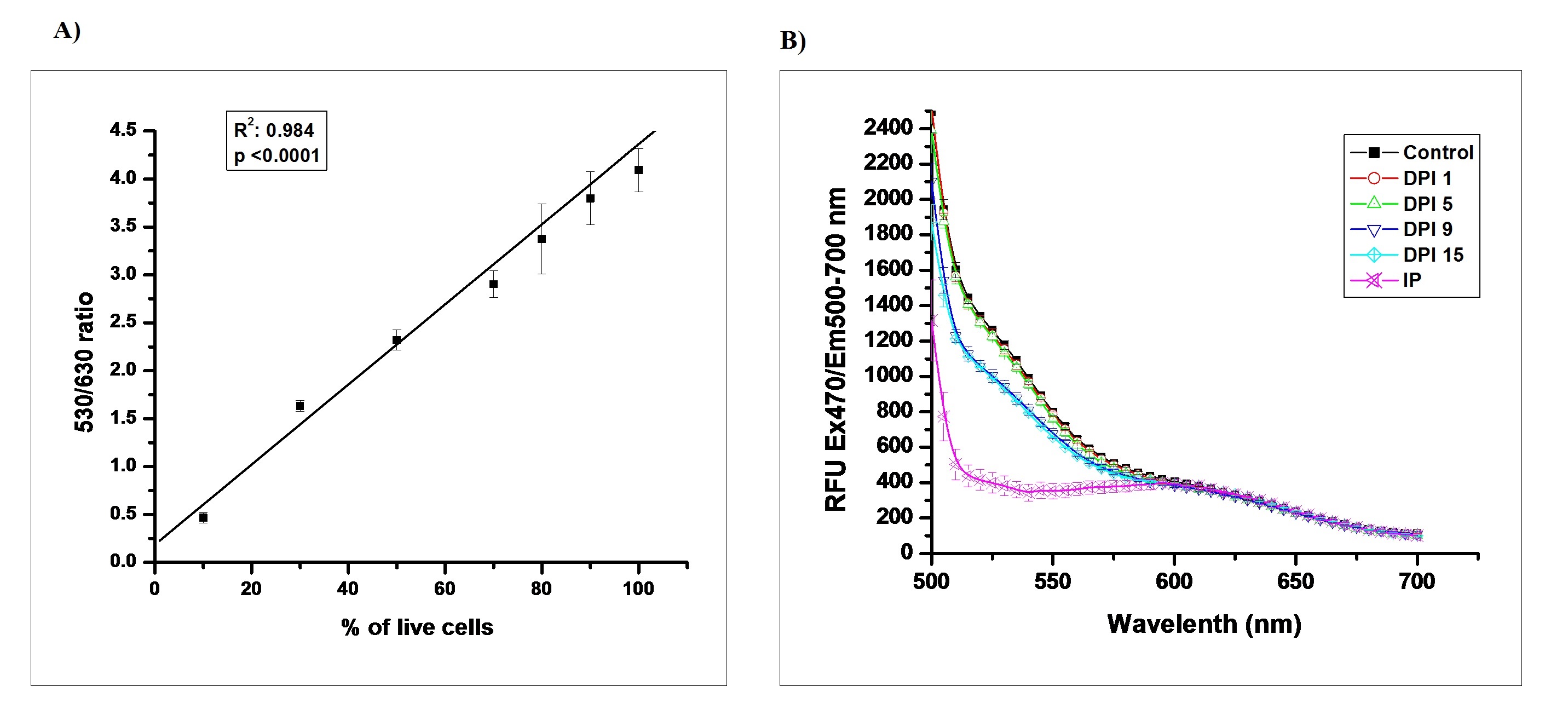

Supplement: S2 Fig — Standardization of Live/dead assay A) Standard plot of % live cell (percentage of live and dead cell obtained by Isopropanol treatment) versus 630/530nm ratio. B) Fluorescence reading of with treated and untreated culture at Ex. 470 and Em. 500-700nm. Assay standardization was done according to manufacture instruction (Invitrogen, Life technology). (JPG) [file pone.0220628.s002.jpg]

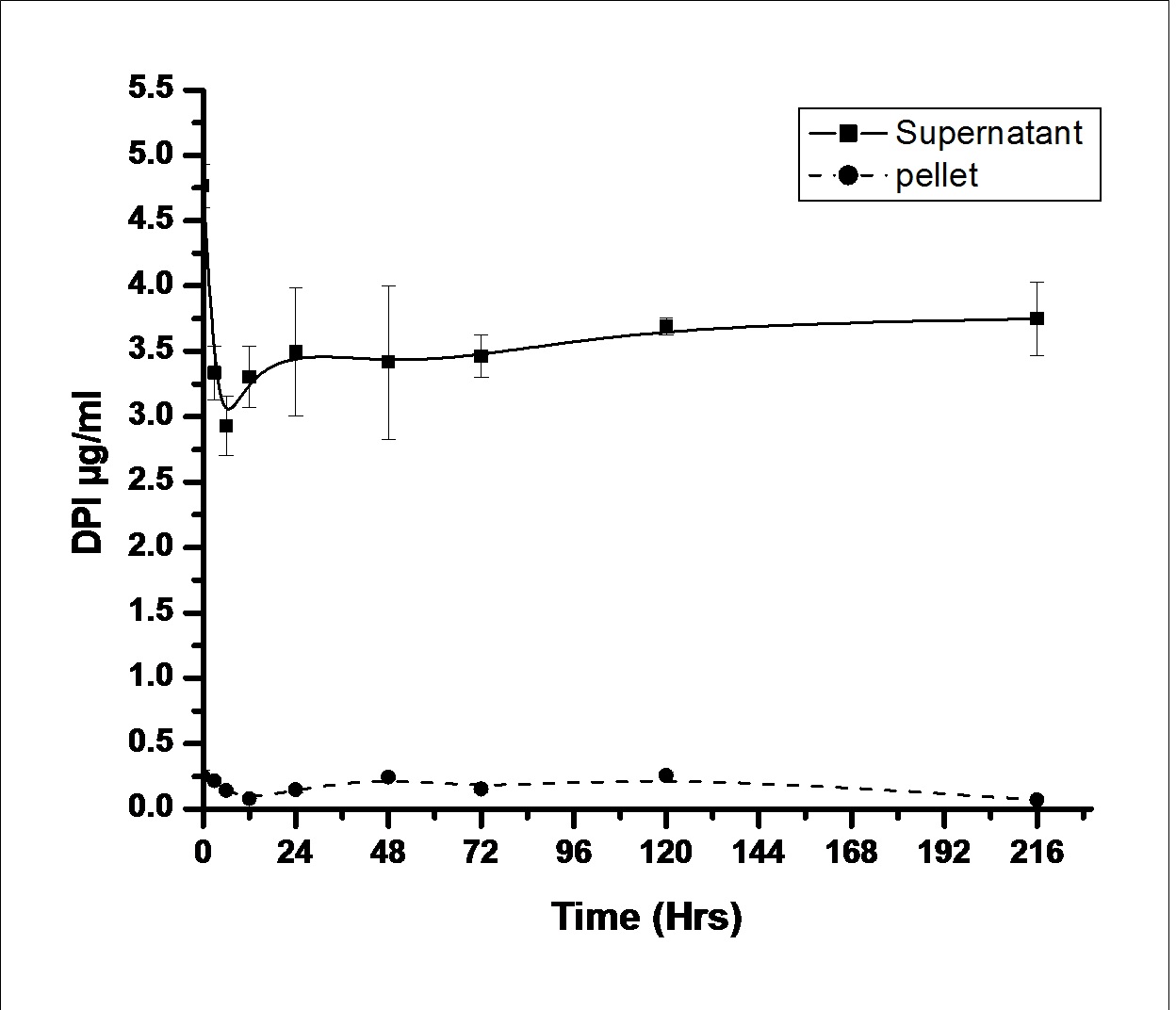

Supplement: S3 Fig — (JPG) [file pone.0220628.s003.jpg]

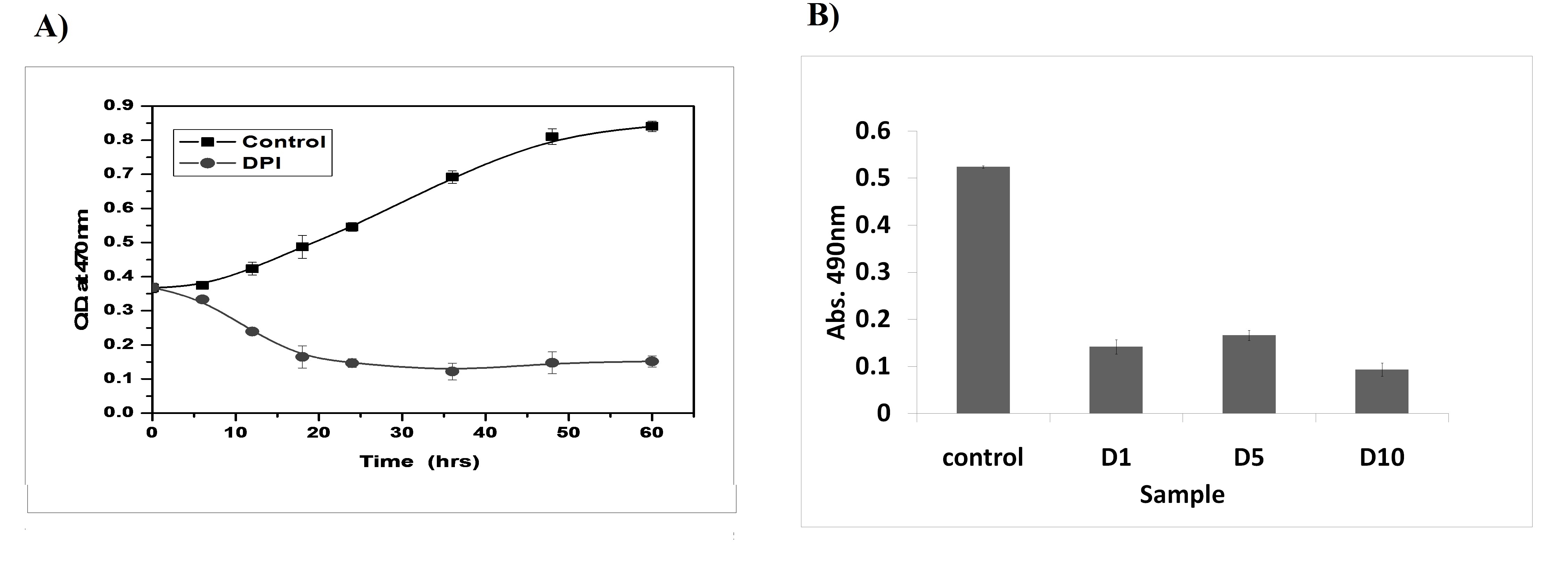

Supplement: S4 Fig — An aliquot of 200 μL of DPI treated bacilli periodically transferred to 96 well plates and incubated with XTT (A) and INT (B) tetrazolium salt. After 20 min incubation with XTT, 60 μM of menadione were added and plate further incubated for 40 mins. The absorbance was measured at 470 nm for XTT reduction assay or 495 nm for the INT reduction assay. Data shown as the mean of three identical results with ±SD. (JPG) [file pone.0220628.s004.jpg]

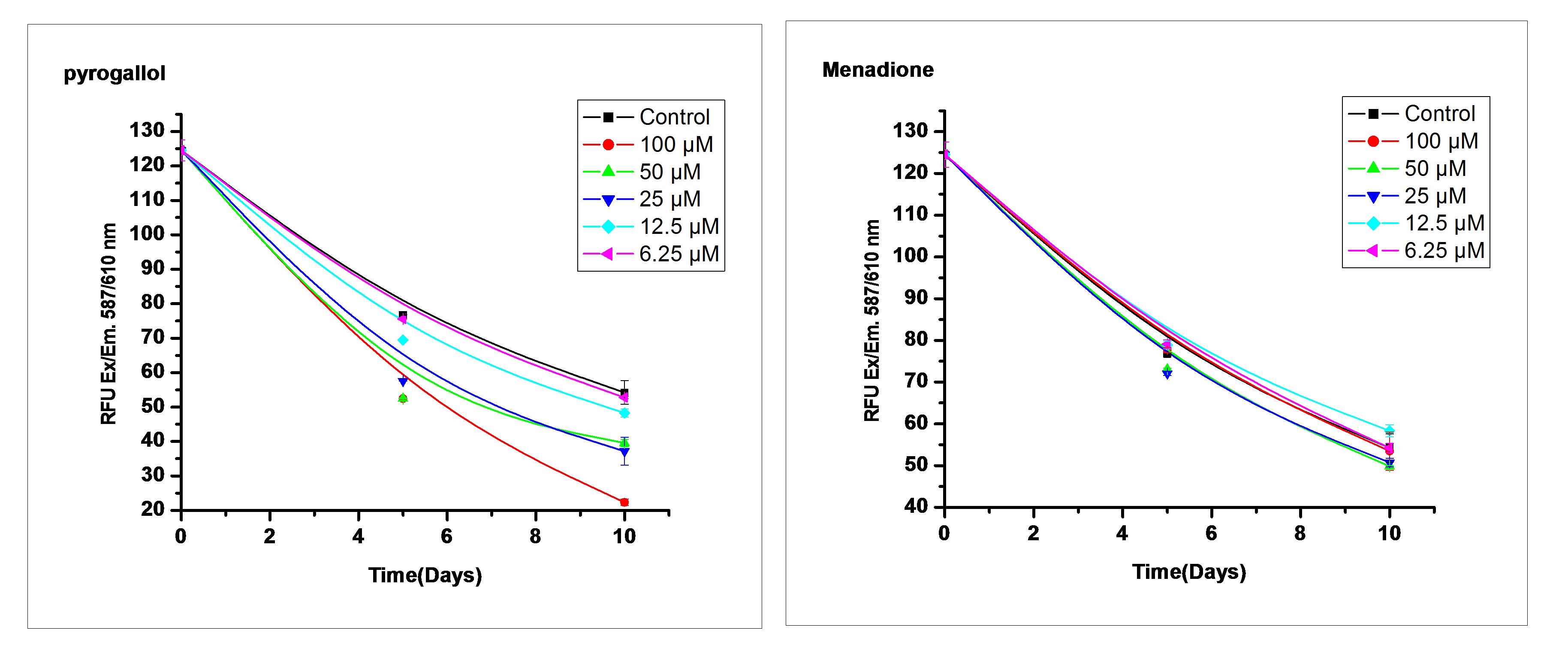

Supplement: S5 Fig — The mycobacterial culture was treated with 4 μg/mL of DPI (OD 600nm = 0.35) for 24–36 h, washed and resuspended in fresh Dubos albumin broth. The various concentration of the Pyrogallol or Menadione were added and incubated for at 37 oC. At various time point growth was measured in the form of fluorescence a Ex/Em. 587/610 nm. Data shown as the mean of triplicate results with ±SD. (JPG) [file pone.0220628.s005.jpg]

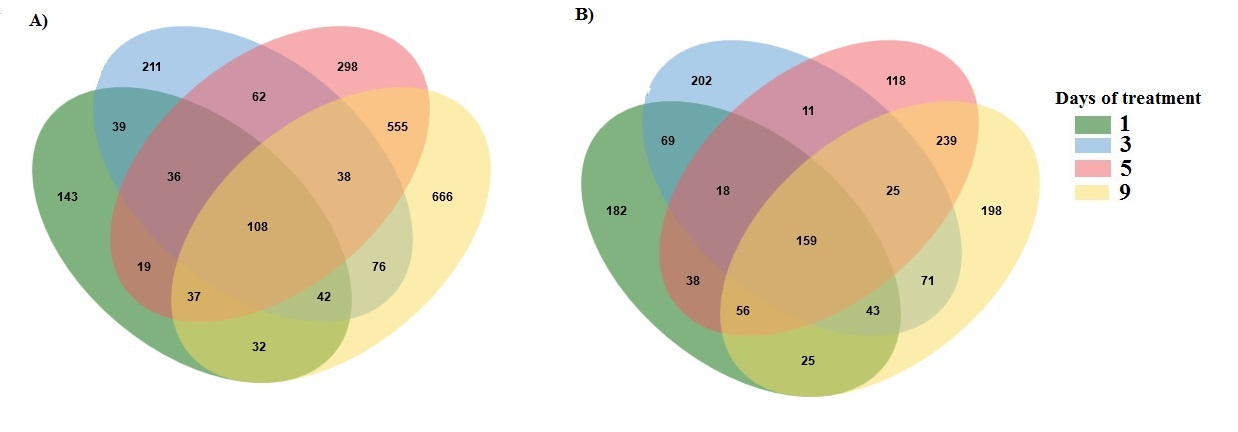

Supplement: S6 Fig — Venn diagram for common feature analysis in A) Positive and B) Negative ion mode analysis. (TIF) [file pone.0220628.s006.tif]

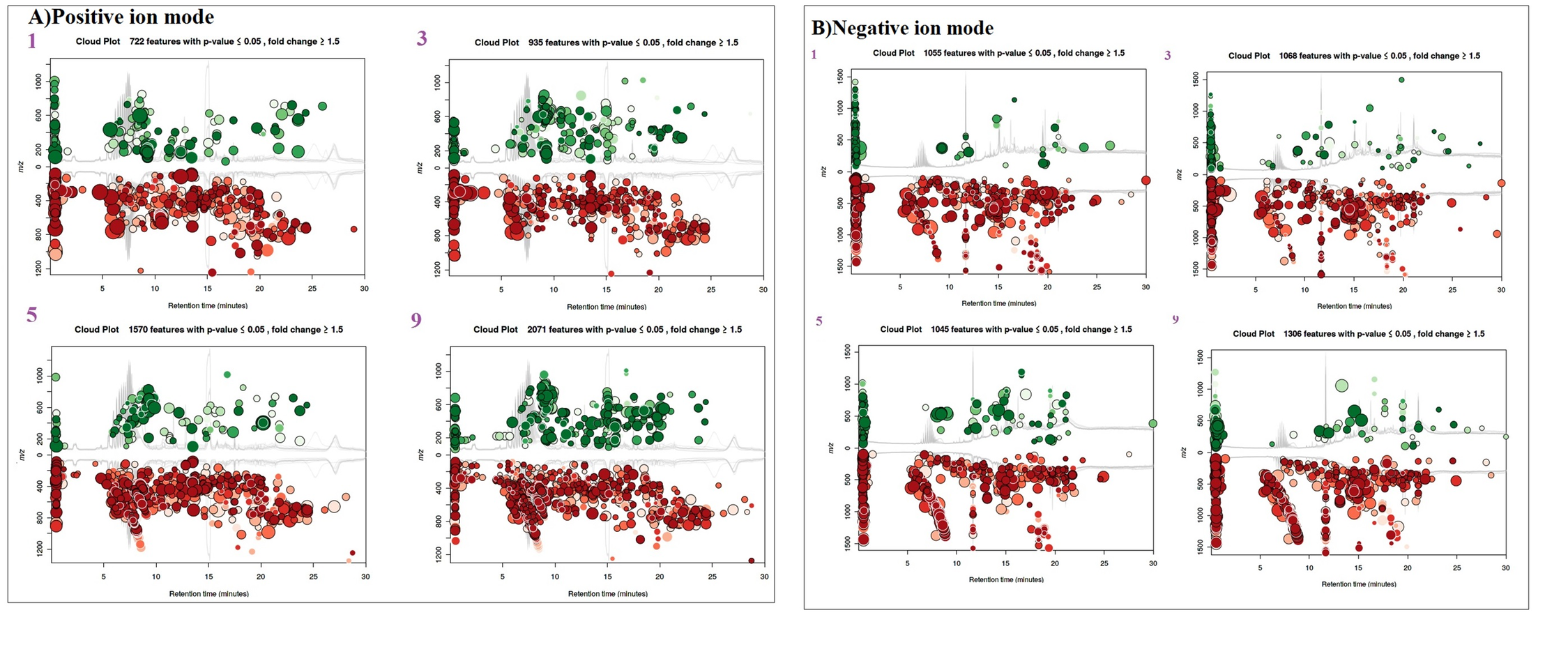

Supplement: S7 Fig — Cloud plots of altered metabolites for each time points by A) Positive and B) Negative ion mode. (TIF) [file pone.0220628.s007.tif]
