# Supplementary material for: Using diphenyleneiodonium to induce a viable but non-culturable phenotype in Mycobacterium tuberculosis and its metabolomics analysis
Source: PLoS One. 2019 Aug 1;14(8):e0220628. doi: 10.1371/journal.pone.0220628 (PMC6675104; doi:10.1371/journal.pone.0220628)
Supplement: S8 Fig — (PDF) [file pone.0220628.s008.pdf]

S8 Fig. Heat map of altered metabolite of DPI treated mycobacteria at 1st , 3rd ,5th and 9th days of treatment

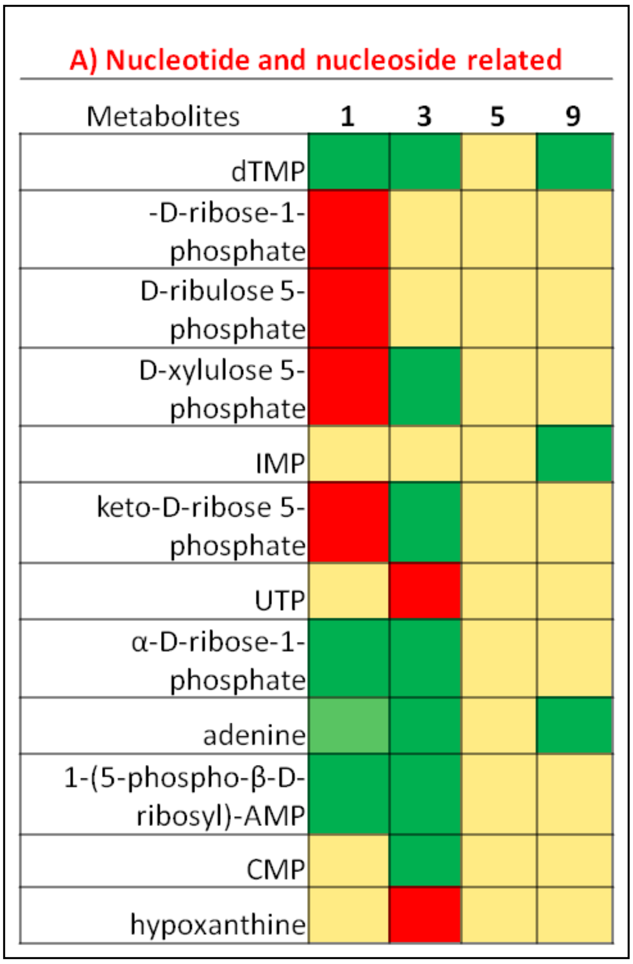

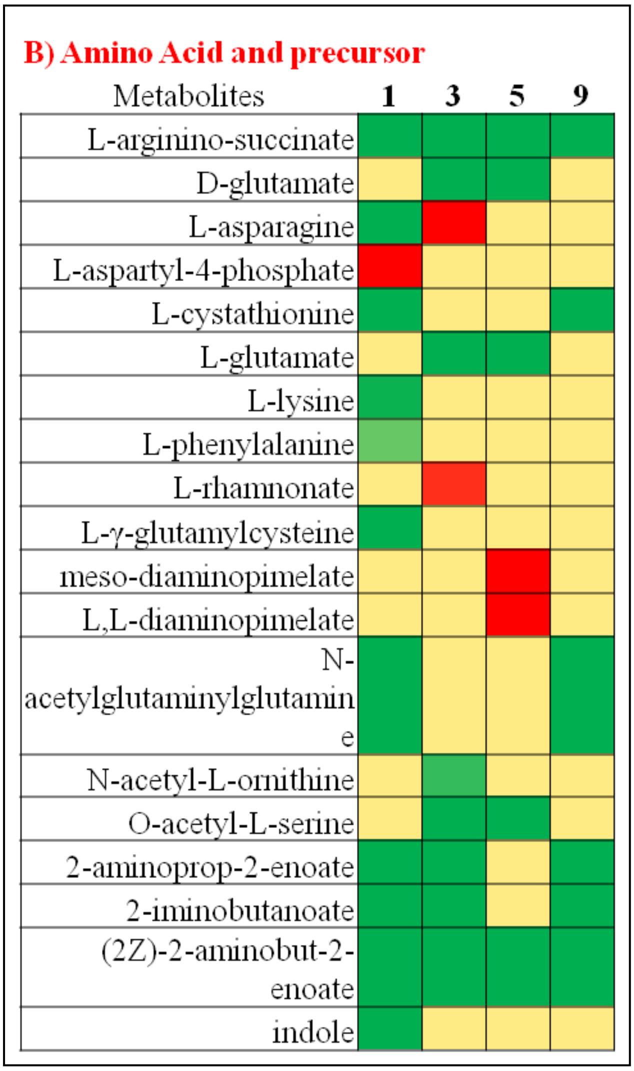

C) Organic acid

Metabolites	1	3	5	9
fumarate				
oxaloacetate				
D-threo-isocitrate				
R)-lactate				
succinate				
succinate semialdehyde				
(R)-lactate				
(S)-2-acetolactate				
citrate				
(S)-lactate				
(S)-malate				
cis-vaccenate				
(2R,3S)-2-methylisocitrate				
(2S)-2-phospholactate				
3-methyl-2-oxobutanoate				
(S)-2-aceto-2-hydroxybutanoate				
(S)-4-hydroxy-2-oxohexanoate				
(R)-2,3-dihydroxy-3-methylbutanoate				
(S)-3-hydroxy-isobutanoate				
2-succinyl-5-enolpyruvyl-6-hydroxy-3-cyclohexene-1-carboxylate				
3-[(3aS,4S,5R,7aS)-5-hydroxy-7a-methyl-1-oxo-octahydro-1H-indene-4-carboxylate				
2-dehydropantoate				
2-oxobutanoate				
8-amino-7-oxononanoate				
(S)-methylmalonate-semialdehyde				
5-enolpyruvyl-shikimate 3-phosphate				

D) Sugar

Metabolites	1	3	5	9
inosine				
chitotriose				
D-glucosamine 1-phosphate				
D-sedoheptulose 7-phosphate				
dTDP-β-L-rhamnose				
glycerol				
sn-glycerol 3-phosphate				
UDP-N-acetyl-α-D-glucosamine				
UDP-α-D-galactose				
UDP-α-D-glucose				
α,α-trehalose				
α-D-glucosamine 6-phosphate				
3-hydroxy-9,10-secoandrosta-1,3,5(10)-triene-9,17-dione				
D-erythro-imidazole-glycerol-phosphate				
dihydroxyacetone phosphate				
UMP				
3-deoxy-D-arabino-heptulosonate-7-phosphate				
2-O-(6-phospho-α-D-mannosyl)-D-glycerate				
2-O-(α-D-glucopyranosyl)-D-glycerate				
2-[2-O-(α-D-mannopyranosyl)-α-D-glucopyranosyl]-3-phospho-D-glycerate				
2-C-methyl-D-erythritol 4-phosphate				
5,6-dimethylbenzimidazole				
5-formamido-1-(5-phospho-D-ribosyl)-imidazole-4-carboxamide				
9α-hydroxyandrosta-1,4-diene-3,17-dione				

E) Lipid and fatty acid

Metabolites	1	3	5	9
L-1-glycero-3-phosphocholine				
octadecanal				
octanoate				
palmitoleate				
phosphocholine				
stearate				
3-oxo-23,24-bisnorchol-4-en-22-oyl-CoA				
3-oxocholest-4-en-26-al				
3-oxocholest-4-en-26-oyl-CoA				

F) Sulfur metabolisms

Metabolites	1	3	5	9
sulfate				
mycothiol				
mycothione				
myo-inositol				
S-methyl-5'-thioadenosine				
dethiobiotin				
glutathioselenol				
L-cystathionine				

G) Energy metabolisms

Metabolites	1	3	5	9
NAD+				
nicotinate				
MoO2-molybdopterin cofactor				
protoporphyrin IX				
