# Supplementary material for: Using diphenyleneiodonium to induce a viable but non-culturable phenotype in Mycobacterium tuberculosis and its metabolomics analysis
Source: PLoS One. 2019 Aug 1;14(8):e0220628. doi: 10.1371/journal.pone.0220628 (PMC6675104; doi:10.1371/journal.pone.0220628)
Supplement: S1 Table — (DOC) [file pone.0220628.s009.doc]

**Table S1: Primers used in this study**

| Sr. No. | Gene | Primers |
| --- | --- | --- |
| 1 | MRA2046 (*Hsp20)* | For: GCTGGAAGACGAGATGAA |
|  |  | Rev: CGAACGAAGGAACCGTA |
| 2 | MRA 1998 (*Ici-lysR)* | For: CATGTTGGTGCGTAAGG |
|  |  | Rev: GACGTCGAGGTGTATGT |
| 3 | MRA_3121 (*Fad13Ligase)* | For: CACGAATCGGTGCATTC |
|  |  | Rev: TCAGTGACCACACCTTC |
| 4 | MRA_0700A (*Hypothetical protein )* | For: CGACTACACCCACAGGT |
|  |  | Rev: ATCCTGGTCACAGTCGT |
| 5 | MRA0473  (*Icl-Lyase)* | For: CTTCTACCGCACCAAGAA |
|  |  | Rev: GATGGCGAGCAGTTGTA |
| 6 | MRA 1175 (*NarG)* | For: CGGAATGACAAGCTGATG |
|  |  | Rev: CACATGCACCTGGTAGTA |
| 7 | MRA 3165  (*Dev-R)* | For: GCGGATATGTCGTCAAAG |
|  |  | Rev: GTCGGTAAGGCCTGATA |
| 8 | MRA 3270  *(L- Co.A saturase)* | For: CTGTCGTTGCCGGATAA |
|  |  | Rev: TGACCGACTTAGCCATAC |
| 9 | MRA 2731 (SigA) | For: GCTGATGACCGAGCTTA |
|  |  | Rev: CTGGATCAGGTCGAGAAA |
